# Treatment of Female Diseases

**Published:** 1861-01

**Authors:** 


TREATMENT OF FEMALE DISEASES.—Dr. Horatio R. Storer, so well known by his excellent Papers on Criminal Abortion in this journal, has opened an establishment for the Treatment of the Diseases of Women, at Blue Hills, near Boston. Professor Storer and Dr. J. M. Warren are the consulting surgeons.

